# Are the Two Schools Alike?

**Published:** 1890-01

**Authors:** Edmund J. Lee

**Affiliations:** Philadelphia


### ARE THE TWO SCHOOLS ALIKE?

EDMUND J. LEE, M. D., PHILADELPHIA.

It has always been understood that the so-called regulars and the homœopathists differed in their ideas of the true method of practicing medicine. Such is the prevalent idea held by the laity in general; such is the common opinion of physicians in general. The so-called regulars claim that they are guided in their practice by "experience," that they use any and every means which experience has taught them may be helpful in relieving suffering humanity. Such is the plausible claim of the regulars; but, in fact, it is not true, for, for the most part, they are guided solely by blind prejudice. They reject with scorn any claims for clinical superiority made by the homœopaths. They reject these claims without any adequate examination. They reject them solely from prejudice. A celebrated surgeon of this city, now deceased, said he knew the *Organon* was not correct, *because he had read it.*

In contrast to the allopathic idea of medical practice, we have that of the homœopath, who proclaims that he is guided, in his use of drugs, by a law; that the use of this law enables him to prescribe with great accuracy, that it enables him to cure promptly and permanently.

In view of the conflicting claims of these two schools of medical practitioners, it is curious to read such statements as the foling:

> "We are surprised and delighted to observe how near Professor Wood's views accord with the great majority of those who call themselves 'homœopathic physicians,' and one has only to scan the literature of the different schools, as the writer has done, to observe how closely they approach each other in their methods, and if it were not for the name it would be difficult to distinguish one from the other."—*N. Y. Medical Times.*

This "surprise and delight" were called forth by an address delivered at Yale College, by Professor Wood. In this address he ridiculed the idea that there could be any law governing the action of drugs. Dr. Wood never enlightened us as to why there could be no law governing this branch of Nature's action; probably he will do this later. There is now, and ever has been, a very marked difference between allopathy and Homœopathy, but there are every year more and more so-called homœopaths whose practice differs little from that of the allopath. It is the so-called "literature" of these eclectic-homœopaths that closely resembles allopathic literature. Genuine Homœopathy differs as much from allopathy as ever it did, and is, as ever before, greatly its superior.

The kind of homœopaths that ape allopathy seem to abound in New York. At a recent meeting of a committee of these so-called homœopaths with the Commissioners of Charities of New York, in regard to Ward's Island Hospital, a Commissioner, one Simmons, told the homœopaths: "You have a homœopathic college, and the young men whom it graduates should be homœopths, if there are any. But it appears that these young graduates, who have been put on the house staff at Ward's Island Hospital have used forty-four pounds of castor oil and twenty-one pounds of magnesia sulph. Dr. Schley, here, is accused of giving large doses of antipyrin and other non-homœopathic remedies." Is it any wonder, in view of such facts, that Professor Wood should declare that homœopaths are either fools or knaves?

If it be true, as the *Times* declares, that the "great majority of those who call themselves homœopathic physicians" are in accord with Professor Wood's views, why then do they call themselves homœopathic physicians? Why do they masquerade under a false name? Dr. Wôod says they do it for the commercial value of the name. Genuine Hahnemannian Homœopathy is a scientific method of practicing medicine. It is not only scientific, but it is also practical and most successful. It not only enables the student of its therapeutics to predict, in advance of any clinical trial, the value of any drug, but it also enables him to prognosticate as to the curability of any given case of disease. It is like a mathematical problem, in that having two or more known quantities we are able to find the unknown. Knowing the symptoms, we can readily find the curative remedy; or, knowing the remedy, we have a helpful guide in our prognosis; or, knowing the remedy, we have a reliable guide for selecting the diet; or, knowing the peculiar cravings of the appetite, we have a reliable aid in choosing the remedy.

Allopathy has no medical views that in any way "accord" with such scientific practice; it is a system of guess-work, as unscientific as it is unsuccessful. In the present epidemic of "La Grippe," now so prevalent here, the success of the old school has been simply ridiculous. Every day we hear of cases of persons dying of "influenza, followed by pneumonia;" a true statement would read, "dying of influenza, followed by Quinine." I have yet to hear of one case dying under the care of a Hahnemannian. About two weeks ago a little boy of eight years was attacked by the influenza; he was at the house of a relative who employs one of the best old-school physicians in this city. This regular saw the little fellow, and advised that he be sent home at once, as he seemed to be on the verge of a serious illness. When the little patient arrived home he complained of headache, pain in back and limbs; his face was very red, the skin very hot, his eyes injected and painful; he wanted to go to bed of his own accord. One dose of Belladonna was given to him; he went to sleep, and awoke in about three hours, got up, dressed himself, and went off to play with the other children. He has not been sick since, nor has he had any more medicine. Was the allopath mistaken in his opinion, or did the one dose of Belladonna cure this child in a few hours? I may add that Belladonna has in the same manner cured at least a dozen patients, in my hands, in the past two weeks.

Genuine Homœopathy is as far in advance of allopathy and Dr. Wood as it ever was. It is only the "literature" and the "views" of the pseudo-homœopaths that in any way accord with the teaching or the practice of the old school.

